# Genetic Variants at Chromosome 9p21 and Risk of First Versus Subsequent Coronary Heart Disease Events: A Systematic Review and Meta-Analysis

**DOI:** 10.1016/j.jacc.2014.01.065

**Published:** 2014-06-03

**Authors:** Riyaz S. Patel, Folkert W. Asselbergs, Arshed A. Quyyumi, Tom M. Palmer, Chris I. Finan, Vinicius Tragante, John Deanfield, Harry Hemingway, Aroon D. Hingorani, Michael V. Holmes

**Affiliations:** ∗ Department of Epidemiology and Public Health, University College London, London, United Kingdom; † Department of Cardiology, The Heart Hospital, University College London NHS Trust, London, United Kingdom; ‡ Genetic Epidemiology Group, Department of Epidemiology and Public Health, Institute of Cardiovascular Science, University College London, London, United Kingdom; § Department of Cardiology, Division of Heart & Lungs, University Medical Center, Utrecht, the Netherlands; ‖ Durrer Center for Cardiogenetic Research, ICIN-Netherlands Heart Institute, Utrecht, the Netherlands; ¶ Department of Medicine, Emory Clinical Cardiovascular Research Institute, Division of Cardiology, Emory University School of Medicine, Atlanta, Georgia; # Division of Health Sciences, Warwick Medical School, University of Warwick, Coventry, United Kingdom; ∗∗ National Institute for Cardiovascular Outcome Research, University College London, London, United Kingdom; †† Department of Surgery, Division of Transplantation, and Center for Clinical Epidemiology and Biostatistics, Perelman School of Medicine, University of Pennsylvania, Philadelphia, Pennsylvania

**Keywords:** coronary heart disease, genomics, incident, subsequent, 9p21, Ch9p21, chromosome 9p21 locus, CHD, coronary heart disease, CI, confidence interval, HR, hazard ratio, MI, myocardial infarction

## Abstract

**Objectives:**

The purpose of this analysis was to compare the association between variants at the chromosome 9p21 locus (Ch9p21) and risk of *first* versus *subsequent* coronary heart disease (CHD) events through systematic review and meta-analysis.

**Background:**

Ch9p21 is a recognized risk factor for a first CHD event. However, its association with risk of subsequent events in patients with established CHD is less clear.

**Methods:**

We searched PubMed and EMBASE for prospective studies reporting association of Ch9p21 with incident CHD events and extracted information on cohort type (individuals without prior CHD or individuals with established CHD) and effect estimates for risk of events.

**Results:**

We identified 31 cohorts reporting on 193,372 individuals. Among the 16 cohorts of individuals without prior CHD (n = 168,209), there were 15,664 first CHD events. Ch9p21 was associated with a pooled hazard ratio (HR) of a first event of 1.19 (95% confidence interval: 1.17 to 1.22) per risk allele. In individuals with established CHD (n = 25,163), there were 4,436 subsequent events providing >99% and 91% power to detect a per-allele HR of 1.19 or 1.10, respectively. The pooled HR for subsequent events was 1.01 (95% confidence interval: 0.97 to 1.06) per risk allele. There was strong evidence of heterogeneity between the effect estimates for first and subsequent events (p value for heterogeneity = 5.6 × 10^−11^). We found no evidence for biases to account for these findings.

**Conclusions:**

Ch9p21 shows differential association with risk of first versus subsequent CHD events. This has implications for genetic risk prediction in patients with established CHD and for mechanistic understanding of how Ch9p21 influences risk of CHD.

The chromosome 9p21 (Ch9p21) locus remains the most widely recognized and replicated genetic risk factor for coronary heart disease (CHD) to date. It was identified through genome-wide association using predominantly case-control studies in which cases had a first CHD event and controls did not 1, 2, 3, 4. Thereafter, studies focusing exclusively on prospective follow-up of individuals without prior CHD confirmed the association of Ch9p21 variants with the risk of *first* CHD events (fatal or nonfatal myocardial infarction [MI], angina, or revascularization) 5, 6, 7, 8. However, conflicting findings have been reported on the association of Ch9p21 variants with risk of *subsequent* CHD events during prospective follow-up of individuals with established CHD, although most of these studies have been small 9, 10, 11, 12, 13.

The question of whether there is a similar association of genetic variants at Ch9p21 with first and subsequent events is important. A difference would imply that assessments of the predictive utility of Ch9p21 variants in individuals without prior CHD should not be extrapolated to those with prevalent disease and, secondly, that (in contrast to a widely-held view) the pathogenesis of first and subsequent CHD events may not be precisely equivalent, with implications for drug development and secondary prevention.

To address this question, we sought to provide more reliable evidence on the association of Ch9p21 genetic variants with first and subsequent CHD events through systematic review and meta-analysis with consideration of potential sources of bias that could account for any differences identified.

## Methods

### Search strategy

Following guidance from PRISMA (14), we searched PubMed and EMBASE from inception to June 30, 2013, for studies reporting on genetic variants at Ch9p21 and CHD. We also searched studies reporting associations of genetic risk scores with CHD, as they may have incorporated data on the Ch9p21 locus. The search strategy thus encompassed terms capturing the genetic locus (9p21 or 9p21.3), genetic risk score, and CHD. The full search terms are provided in the Online Appendix.

Retrieved papers were screened for those reporting association of Ch9p21 variants with incident fatal/nonfatal CHD events during follow-up. Thus, prospective cohorts and nested case-control or -cohort studies were included, whereas case-control or cross-sectional studies of Ch9p21 and CHD were omitted, as were editorials and reviews. We hand-searched bibliographies of included papers, prior meta-analyses, and review papers to find studies that were not captured by the original search. We did not impose a language restriction.

Two authors (R.S.P. and M.V.H.) extracted information and any discrepancies were resolved by consensus. The following information was extracted: 1) clinical setting (general population cohorts followed-up for first CHD events or cohorts of patients with CHD followed-up for subsequent CHD events); 2) reported outcome(s), including whether outcome ascertainment was reported as adjudicated; 3) sample size and number of events; 4) duration of follow up; 5) ethnicity; 6) genetic effect estimates; 7) covariates used for any adjustment; and 8) the SNP reference sequence (rs) number and other quality control indexes (e.g., reporting of deviation from Hardy Weinberg equilibrium, genotyping call rate, and genotyping technique).

### Outcomes

The primary analysis investigated a pre-specified composite CHD outcome (encompassing any of fatal or nonfatal MI, angina, or revascularization) and was limited to individuals of European ancestry. For studies of individuals with established CHD, the composite CHD outcome was the same, with the exception that all-cause mortality was included, on the assumption that vascular events are the most common underlying cause of death following a previous CHD event 15, 16. Where the reported outcome deviated from these pre-specified definitions, the outcome closest to the pre-specified composite outcome was included. When a study reported a composite outcome that included stroke, this was also included in the analysis. When a study reported only 1 of the composite outcomes, it was included in this analysis (so that representation was made from all studies when possible). The constituents of the CHD composite for each study are listed in Table 1.

**Table 1:** Characteristics of Eligible Studies

First Author, Cohort Name (Ref. #)	Number of Individuals	Sampling Frame	Ethnicity,% Caucasian	Men, %	Age, Mean ± SD, yrs	Duration of Follow-Up (yrs)	Outcomes Included in “CHD Event” Composite	Outcome Ascertainment	Outcome Adjudicated
Studies Reporting First CHD Events in Individuals Predominantly Free From Established CVD									
Ye et al., Bruneck (6)	769	Population based	100	50	63 ± 11	10.0	Fatal/nonfatal MI, revascularization	Medical record review, study protocol	Not stated
Dutta et al., EPES (8)	1,095	Population based	100	34	80 ± 9	20.0	Fatal CHD	Death certificates, NDI, ICD codes	Not stated
Lluis-Ganella et al., REGICOR (30)	2,351	Population based	100	48	54 ± 11	9.8	MI, angina, revascularization, fatal CHD	Interval follow-up, medical records, state and national mortality registers	Committee
Talmud et al., Northwick Park (5)	2,742	General practices	100	100	56 ± 3	15.0	Fatal/nonfatal CHD, revascularization	GP, hospital, coroner’s office. Independent review.	Not stated
Vaarhorst et al., CAREMA (36)	2,963	Municipal registries	100	60	47 ± 7	12.1	MI, unstable angina, fatal CHD	Linked hospital records and national death records	Not stated
Lluis-Ganella, Framingham (30)	3,537	Population based	100	44	56 ± 9	13.3	MI, angina, revascularization, fatal CHD	Interval follow-up, medical records	Committee
Franceschini et al., CHS (26)	3,978	Population based	100	40	73 ± 6	11.5	MI, fatal CHD, revascularization	Annual visits, records	Committee and physician review panel
Wahlstrand et al., NORDIL (34)	5,262	Hypertensive patients	100	50	60 ± 6	4.5	MI, revascularization	6-month visits, records	Committee
Dehghan et al., Rotterdam (24)	7,983	Population based	100	40	69.5 ± 9	9.5	Fatal/nonfatal MI, revascularization	Record linkage, medical record review, local death records	2 research physicians independently coded events and 1 expert in CVD made final decision
Franceschini et al., ARIC (26)	10,247	Population based	100	45	54 ± 6	15.7	MI, fatal CHD, revascularization	3-year follow up contact, records	2 physicians and differences adjudicated
McPherson et al., Copenhagen City Heart Study (1)	10,578	Population based	100	44	58 ± 15	15.0	Ischemic CV event	Registry ICD9	Not stated
Franceschini et al., Women’s Health Initiative (26)	12,392	Population based	100	0	67 ± 7	9.1	MI, fatal CHD, revascularization	Self-report and medical record review	Not stated
Paynter et al., Women's Health Study (32)	22,129	Women age ≥45 yrs	100	0	53 ± 5	10.2	MI, revascularization, death	Medical record review	Committee
Tikkanen et al., FINRISK/ Health 2000 (33)	24,124	Population based	100	46	48 ± 12	12.0	MI, unstable angina, revascularization, fatal CHD	Finnish hospital and death registers	Not stated
Gransbo et al., MALMO DCS (28)	24,777	Population based	100	38	58 ± 8	11.7	MI, revascularization or death	Registry linkage, ICD10	Not stated
Karvanen et al., MORGAM (7)	33,282	Population based	100	90	58 ± 8	5.0	Fatal/nonfatal MI, fatal CHD, unstable angina, revascularization, death	Questionnaire, ICD codes, hospital discharge register, Register of Causes of Death	Committee

Studies Reporting Subsequent CHD Events in Individuals With Established CHD									
Dutta et al., EPES (8)	478	Population-based cohort with physician-diagnosed or suspected MI and/or angina	100	34	80 ± 9	20.0	Fatal CHD	Death certificates, NDI, ICD codes	Not stated
Gioli-Pereira et al., MASS II (27)	496	Patients with angiographically-documented proximal multivessel coronary stenosis >70% and documented ischemia	100	67	60 ± 9	5.0	All-cause mortality	6 monthly visits	Not stated
Andreassi et al., GENECOR (17)	498	Patients with MI or angina, ≥1 vessel disease at coronary angiography (>50% lumen reduction)	100	87	57 ± 8	6.9	MI, revascularization, fatal CHD	Telephone interview, medical records, death certificates	Not stated
Gong et al., INFORM (10)	557	Patients with CAD and hypertension	100	64	63 ± 12	2.0	All-cause mortality, rehospitalization for MI, heart failure, chest pain, or revascularization	Interview, records, SSDI	Not stated
Horne et al., Intermountain 1B (11)	1,014	Patients undergoing angiography with CAD: ≥1 vessel disease at coronary angiography (≥70% stenosis)	90	73	62 ± 10	3.6	Nonfatal MI or all-cause mortality	Hospital database, SSDI, death certificates	Not stated
Virani et al., TexGen (CABG) (13)	1,176	Patients undergoing CABG surgery	100	79	65 ± 10	3.2	Fatal/nonfatal MI	Annual telephone survey and medical records and state records	Not stated
Ardissino et al., IGSEMI (9)	1,508	Patients undergoing angiography with CAD: ≥1 vessel disease at coronary angiography (≥70% stenosis)	100	86	41 ± 6	10.0	CVD, MI, revascularization	Death certificates, source data verification	2 cardiologists and 3rd arbitrating
Horne et al., Intermountain 1A (11)	1,748	Patients undergoing angiography with CAD: ≥1 vessel disease at coronary angiography (≥70% stenosis)	85	78	63 ± 10	6.7	Nonfatal MI, all-cause mortality	Hospital database, SSDI, death certificates	Not stated
Hoppmann et al., German Stent Study (18)	2,028	Patients with symptoms/evidence of myocardial ischemia and ≥50% stenosis on angiography	100	78	66 ± 10	3.0	All-cause mortality, MI, target lesion revascularization	Scheduled visit or telephone interview	Not stated
Virani et al., TexGen (ACS) (13)	2,067	Patients presenting to the hospital with ACS	100	74	63 ± 11	3.2	Fatal/nonfatal MI	Annual telephone survey and medical and state records	Not stated
Wauters et al., GRACE (34)	2,099	Patients admitted to the hospital with ACS	100	72	66 ± 10	5.0	MI	Telephone, visit, medical records, linkage	Not stated
Gong et al., INVEST-GENES (10)	2,364	Patients with CAD and essential hypertension requiring drug therapy who were age ≥50 yrs	100	54	69 ± 10	2.8	MI, stroke, all-cause mortality	RCT, events reporting, records	Committee
Patel, Emory, unpublished data, 2013	2,641	Patients undergoing coronary angiography for known or suspected CAD	100	69	64 ± 11	3.6	MI or all-cause mortality	Telephone, medical records, SSDI, death certificates	2 cardiologists and 3rd arbitrating
Patel, Cleveland Clinic (23)	2,702	Patients undergoing coronary angiography for known or suspected CAD	100	72	63 ± 10	2.9	Fatal/nonfatal MI	Source documentation	2 cardiologists and 3rd arbitrating
Tragante et al., SMART, unpublished data, 2013	3,788	Consecutive patients newly referred to the hospital with atherosclerotic cardiovascular disease	100	81	56.5 ± 12	5.0	MI, stroke, all-cause mortality	Questionnaire, general practitioner, hospital discharge letters	Committee

ARIC = Atherosclerosis Risk in Communities Study; CABG = coronary artery bypass graft; CAD = coronary artery disease; CAREMA = The Cardiovascular Registry Maastricht; CCHS = Copenhagen City Heart Study; CHD = coronary heart disease; CHS = Cardiovascular Health Study; CVD = cardiovascular disease; EPES = Established Populations for Epidemiological Study; GENECOR = Genetic Mapping for Assessment of Cardiovascular Risk; GP = general practitioner; GRACE = Global Registry of Acute Coronary Events; ICD = *International Classification of Diseases*; IGSEMI = Italian Genetic Study of Early onset MI; INFORM = Investigation of Outcomes From Acute Coronary Syndromes Study; INVEST = International Verapamil SR Trandolapril Study; MALMO DCS = Malmo Diet and Cancer Study; MASS II = Medical, Angioplasty or Surgery Study II; MI = myocardial infarction; MORGAM = MOnica Risk, Genetics, Archiving, Monograph; NDI = National Death Index; NORDIL = Nordic Diltiazem study; REGICOR = Registre Gironí del Cor; SMART = Secondary Manifestation of ARTerial disease; SSDI = Social Security Death Index; WHI = Women's Health Initiative.

In addition to the primary composite outcome, we investigated associations with the following components separately: 1) MI (comprising fatal/nonfatal MI); 2) all-cause mortality; and 3) coronary revascularization. Additionally, we investigated the association of Ch9p21 variants with the following subsidiary outcomes: 1) MI or all-cause mortality; 2) MI, all-cause mortality, unstable angina, revascularization, or hospitalization; and 3) MI, all-cause mortality, unstable angina, revascularization, hospitalization, or peripheral artery disease.

### Analysis

We analyzed data from population-based cohorts of individuals without prior CHD separately from prospective studies of clinical cohorts of patients with established CHD (prior MI and/or coronary artery disease defined on the basis of prior revascularization or angiography). Incident events in the population-based cohorts were termed “first events,” whereas those in clinical cohorts were termed “subsequent events.” We used hazard ratios (HRs) per risk allele of Ch9p21 as the measure of effect.

For 6 studies 2, 5, 13, 17, 18, 19 that reported effect estimates separately for heterozygous (1 risk allele vs. none) and homozygous (2 risk alleles vs. none) comparisons, we generated a per-risk allele estimate by meta-analyzing the 2 values together. This was done by halving the log HR and corresponding SE for the homozygous comparison and pooling with corresponding values reported for the heterozygous comparison through fixed-effects meta-analysis. Studies that only reported a recessive model (0 or 1 risk alleles vs. 2) were not incorporated into the meta-analysis.

Study-specific log HRs and the corresponding SEs were pooled using fixed and random effects meta-analysis, and between-study heterogeneity was quantified using the I^2^ statistic.

Heterogeneity in the association of Ch9p21 with risk of first or subsequent CHD events was tested using the Altman and Bland test for interaction (20).

### Subgroup and sensitivity analyses

#### Identification of Heterogeneity by Subgroup Analyses

We identified sources of heterogeneity by conducting subgroup analyses of the association of Ch9p21 variants with CHD events using the following pre-specified categories: 1) mean age per study (<60 or ≥60 years); 2) proportion male (<50% or ≥50%); 3) duration of follow-up; 4) sample size of cohort; 5) whether outcome ascertainment was adjudicated or not; 6) rs# of SNP used to genotype Ch9p21; and genomic indexes including 7) HWE; 8) genotype platform; and 9) call rate.

Differences in the association between strata and risk of CHD events were tested using the chi-square test for heterogeneity. With 9 subgroup analyses conducted for both first and subsequent CHD events (a total of 18 tests for interaction), the Bonferroni-adjusted p value for deviation from the null hypothesis of no heterogeneity was 0.05/18 = 0.003. For subgroups that showed strong evidence of heterogeneity (p < 0.001), these were incorporated into a meta-regression analysis on the association of Ch9p21 variants with CHD events to examine if their incorporation reduced the heterogeneity (as measured by I^2^) and, therefore, could explain between-study differences in the association of Ch9p21 variants with CHD events.

#### Subsidiary Analysis

In addition to the outcome analyses in the preceding text, which were limited to individuals of European ancestry, we also investigated the association between Ch9p21 and CHD events according to different ethnic groups.

### Assessment of bias

We estimated small-study bias through visual inspection of funnel plots and formally quantified it using Egger’s test. To examine the influence of each individual study, we repeated meta-analyses excluding each study at a time. Potential for survival bias was examined by comparing risk allele frequencies in cohorts predominantly free from CHD and cohorts of patients with established CHD. Evidence for index event bias was sought by examining reported differences in covariate and risk factor distributions by Ch9p21 genotype.

### Estimation of power

Using: 1) the estimate derived from the per-risk allele association of Ch9p21 variants with first events; 2) the pooled event rate of subsequent events in the cohorts set in individuals with established CHD; and 3) a minor allele frequency of the most commonly used SNP rs10757278 of 50% (HapMap Europeans, available at dbSNP [21]), we calculated the power to detect an association of Ch9p21 variants with subsequent CHD events, using a 2-sided alpha of 0.05. This calculation was conducted to estimate the power to detect the same HR for subsequent events as that for first CHD events (HR: 1.19), and also a more modest HR (HR: 1.10). Calculations were performed using the online Genetic Power Calculator (22).

All analyses were conducted using Stata version 13.1 (StataCorp, College Station, Texas).

## Results

The literature search retrieved 327 papers, of which 25 1, 5, 6, 7, 8, 9, 10, 11, 13, 17, 18, 23, 24, 25, 26, 27, 28, 29, 30, 31, 32, 33, 34, 35, 36 satisfied our inclusion criteria, reporting data from 33 cohorts (Online Fig. S1). To this we added data that was unpublished at the time from 2 cohorts (Emory [Patel et al., June 2013]; SMART, Second Manifestations of ARTerial disease [Tragante et al., June 2013]). Three studies including data from 4 cohorts 25, 29, 31 reported genetic results in a recessive format and were not included in genetic analyses. Thus, a total of 31 prospective cohorts of 193,372 individuals with 20,100 CHD events were included in our analysis (Table 1).

Sixteen cohorts of 168,209 individuals without prior CHD were followed-up for a first CHD event. Fifteen cohorts of 25,163 individuals with established CHD were followed-up for subsequent CHD events. One study (8) reported data separately for those with and without established CHD and, therefore, contributed to both the analysis of Ch9p21 with first and subsequent CHD events, and 1 study (13) reported data separately for those with prior coronary artery bypass grafting or acute coronary syndrome.

Adjudicated outcomes ascertainment was reported for 8 of 16 first event studies and for 5 of 15 subsequent event studies (Table 1).

The weighted mean age per study was 57 years (range 47 to 80 years) for first and 62 years (range 41 to 80 years) for subsequent event studies (Table 2). Forty-five percent (range 0% to 100%) and 73% (range 34% to 87%) of study participants were male for first and subsequent event studies, respectively. Median follow-up was for 11.6 years (range 4.5 to 20.0 years) for studies reporting first events and 3.6 years (range 2 to 20 years) for studies reporting subsequent events. Six SNPs were used to genotype the Ch9p21 locus, with the majority of studies (22) using rs10757278 or rs1333049 (Online Table S1) and the remainder using rs10757274, rs133040, rs2383206, or rs4977574. All SNPs except 1 (rs1333040) were in strong linkage disequilibrium with each other (R^2^ > 0.8) (Online Fig. S2). Each study conducted the analysis using the Ch9p21 risk allele as the exposure (Online Table S2). SNPs were in Hardy Weinberg equilibrium (p > 0.01), with a call rate ≥95% in all studies in which this information was recorded (Online Table S1).

**Table 2:** Comparison of Studies Investigating First and Subsequent CHD Events

	First CHD Events	Subsequent CHD Events
Studies	16	15
Participants	168,209	25,163
Events	15,664	4,436
Weighted mean age, yrs	57 (47–80)	62 (41–80)
Male, %	45 (0–100)	73 (34–87)
Median duration of follow-up, yrs	12 (5–20)	4 (2–20)
Studies with adjudicated outcomes	8 (50)	5 (33)

Values are n, mean or median (range), or n (%).

### Association of Ch9p21 variants with first and subsequent CHD events

**Figure 1:**
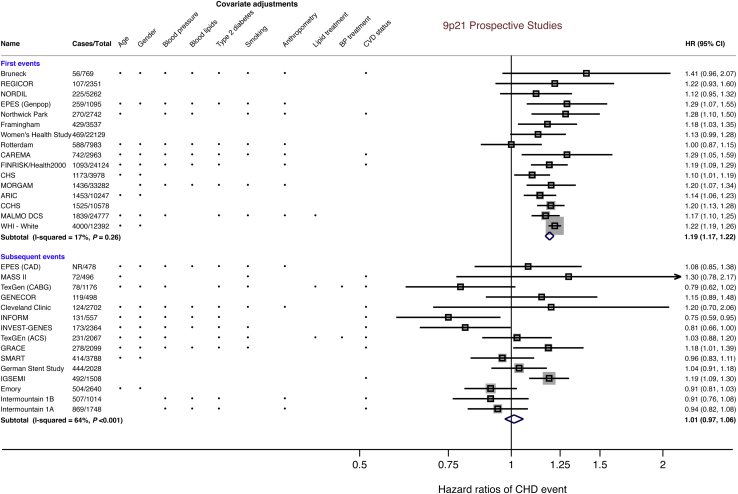
Association of Ch9p21 (Per Risk Allele) With First and Subsequent CHD Events During Prospective Follow-Up Forest plot demonstrating study-specific and pooled hazard ratios between Ch9p21 and risk of incident coronary heart disease (CHD) events in general populations (first events) and CHD populations (subsequent events). Covariate adjustments for each study are also provided. TexGen reported data separately for individuals with previous acute coronary syndrome (ACS) (TexGen [ACS]) or coronary artery disease (TexGen [coronary artery bypass graft (CABG)]). Intermountain reported 2 different datasets (a test set [Intermountain 1A] and replication set [Intermountain 1B]). ARIC = Atherosclerosis Risk in Communities Study; CAREMA = The Cardiovascular Registry Maastricht; CCHS = Copenhagen City Heart Study; CHS = Cardiovascular Health Study; CI = confidence interval; EPES = Established Populations for Epidemiological Study; GENECOR = Genetic Mapping for Assessment of Cardiovascular Risk; GRACE = Global Registry of Acute Coronary Events; HR = hazard ratio; IGSEMI = Italian Genetic Study of Early onset MI; INFORM = Investigation of Outcomes From Acute Coronary Syndromes Study; INVEST = International Verapamil SR Trandolapril Study; MALMO DCS = Malmo Diet and Cancer Study; MASS II = Medical, Angioplasty or Surgery Study II; MORGAM = MOnica Risk, Genetics, Archiving, Monograph; NORDIL = Nordic Diltiazem study; REGICOR = Registre Gironí del Cor; SMART = Secondary Manifestation of ARTerial disease; WHI = Women's Health Initiative.

Almost all studies adjusted for at least 1 cardiovascular risk factor in the analysis of the association of Ch9p21 variants with incident CHD events (Fig. 1). In 16 studies with 15,664 first CHD events, these variants were associated with a per-risk allele pooled HR of 1.19 (95% confidence interval [CI]: 1.17 to 1.22) for first CHD events using fixed-effects (Fig. 1) and an HR of 1.18 (95% CI: 1.15 to 1.21) using random-effects meta-analysis.

In 15 studies with 4,436 subsequent CHD events in patients with established CHD, the pooled per-risk allele HR was 1.01 (95% CI: 0.97 to 1.06) using fixed-effects and 0.99 (95% CI: 0.92 to 1.07) using random-effects meta-analysis.

There was strong evidence of a differential association of Ch9p21 variants with first events in initially healthy individuals without prior CHD versus subsequent CHD events in patients with established CHD (p values for heterogeneity = 5.6 × 10^−11^ and 1.6 × 10^−5^ for comparison of estimates derived from fixed- and random-effects meta-analysis, respectively). A power calculation indicated >99% power to detect a similar HR (HR: 1.19) for subsequent as for first CHD events at a 2-sided alpha value of 5%. The corresponding power to detect a more modest HR for subsequent CHD events of 1.10 was 91%.

### Subgroup analyses and sources of heterogeneity

The association of Ch9p21 variants with first CHD events was consistent across studies, and the heterogeneity between studies was accordingly low (I^2^ = 17%, 95% CI: 0% to 54%) (Fig. 1). In contrast, there was moderate heterogeneity of effect estimates among studies of the association of Ch9p21 variants with subsequent CHD events (I^2^ = 64%, 95% CI: 37% to 79%). Removal of 1 study (Italian Genetic Study of Early Onset MI), which had a considerable influence on the summary estimate for subsequent events (Online Fig. S3), yielded a revised summary HR of 0.97 (95% CI: 0.92 to 1.02), and the I^2^ diminished to 45%.

**Figure 2:**
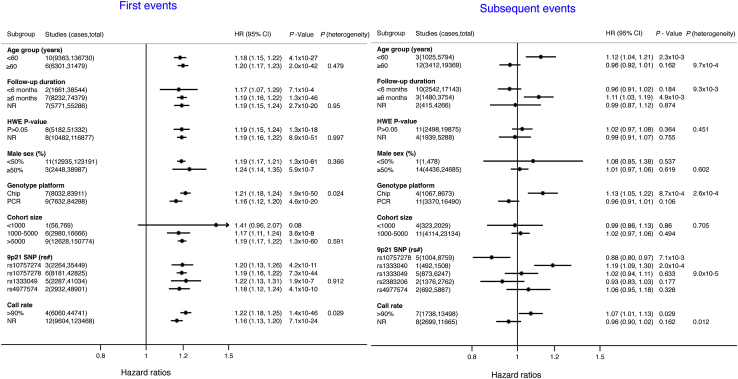
Subgroup Analysis of the Association of Ch9p21 (Per Risk Allele) With First and Subsequent CHD Events Subgroups were chosen a priori. The p value for heterogeneity was obtained from the chi-square test. HWE = Hardy Weinberg Equilibrium; NR = not reported; PCR = polymerase chain reaction; other abbreviations as in Figure 1.

In subgroup analysis, estimates for the association between Ch9p21 variants and first CHD events were similar between subgroup strata (Fig. 2) and no p values for heterogeneity surpassed our Bonferroni-adjusted value (of p < 0.003). In contrast, we identified strong evidence for a differential effect of Ch9p21 on risk of subsequent CHD events by the SNP used to genotype Ch9p21 (p value for heterogeneity = 9 × 10^−5^), mean age per study (p = 9.7 × 10^−4^), and genotype platform (p = 2.6 × 10^−4^). When these variables were entered into a meta-regression term, inclusion of the SNP genotyped had the greatest influence in the heterogeneity statistic, diminishing it from moderate to low, with the corresponding I^2^ statistic falling from 64% to 33% (Online Table S3).

### Association of Ch9p21 variants with individual and composite cardiovascular outcomes

**Figure 3:**
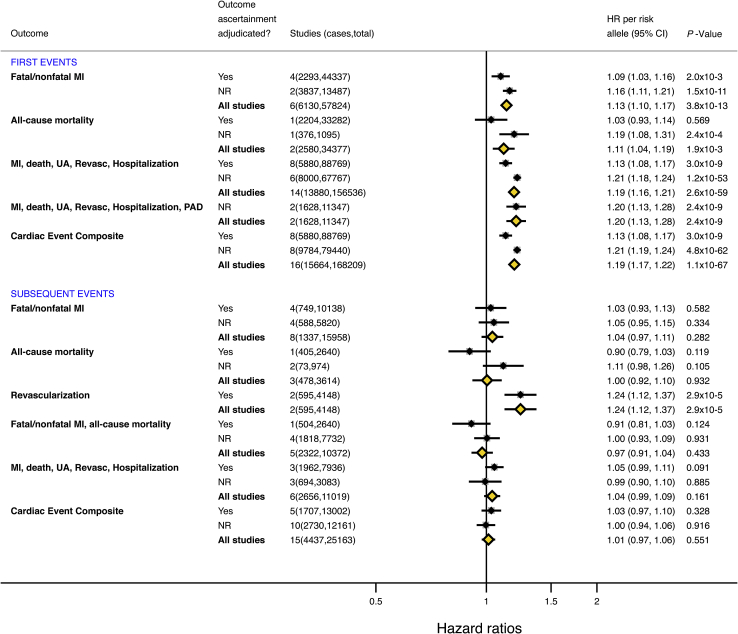
Association of Ch9p21 With Individual and Composite Cardiovascular Outcomes Each outcome is stratified by whether the studies reported adjudication of outcome ascertainment (as reported in Table 1). MI = myocardial infarction; Revasc = revascularization; UA = unstable angina; other abbreviations as in Figure 1.

We also investigated the association of Ch9p21 with cardiovascular outcomes separately and with subsidiary composite endpoints with differing component outcomes (Fig. 3, Online Table S4). In population-based cohorts of individuals without prior CHD, Ch9p21 variants showed consistent associations with all of the individual and subsidiary composite outcomes studied, including first fatal/nonfatal MI (6,130 events in 6 cohorts; HR: 1.13 per risk allele; 95% CI: 1.10 to 1.17) and all-cause mortality (2,580 events in 2 cohorts; HR: 1.11; 95% CI: 1.04 to 1.19). For the composite outcomes, Ch9p21 variants were associated with first MI, death, unstable angina, revascularization, or hospitalization (13,880 events in 14 cohorts; HR: 1.19; 95% CI: 1.16 to 1.21) and a composite that included peripheral artery disease (1,628 events in 2 cohorts; HR: 1.20; 95% CI: 1.13 to 1.28). Of note, most associations were similar when stratified according to whether the outcome was adjudicated or not. The 1 notable exception was the association of Ch9p21 with first CHD events, which was weaker (but remained significant) in studies with adjudicated events (HR: 1.13; 95% CI: 1.08 to 1.17) compared with studies with nonadjudicated events (HR: 1.21; 95% CI: 1.19 to 1.24).

In contrast, Ch9p21 showed inconsistent associations with subsequent events when studies reported outcomes separately or as subsidiary composite endpoints comprising different combinations of outcomes. There was no association of Ch9p21 variants with subsequent fatal/nonfatal MI (1,337 events in 8 cohorts; HR: 1.04 per risk allele; 95% CI: 0.97 to 1.11) or all-cause mortality (478 events in 3 cohorts; HR: 1.00; 95% CI: 0.92 to 1.10), and the estimates did not differ among studies with adjudicated versus nonadjudicated outcomes. However, an association was identified between the risk allele at Ch9p21 and revascularization (595 events in 2 cohorts; HR: 1.24; 95% CI: 1.12 to 1.37). For the composite outcomes, Ch9p21 variants did not show association with subsequent fatal/nonfatal MI or all-cause mortality (2,322 events in 5 cohorts; HR: 0.97; 95% CI: 0.91 to 1.04) or for a composite of subsequent MI, all-cause mortality, unstable angina, revascularization, or hospitalization (2,656 events in 6 cohorts; HR: 1.04; 95% CI: 0.99 to 1.09) (Fig. 3).

### Risk of bias

**Figure 4:**
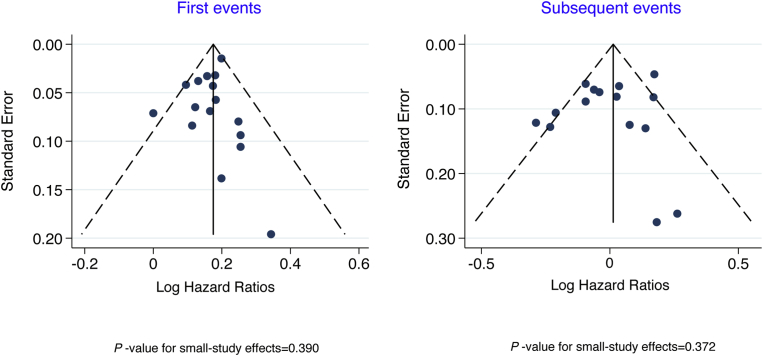
Funnel Plots of the Association of Ch9p21 (Per Risk Allele) With First and Subsequent CHD Events Both funnel plots appeared symmetrical, supported by formal statistical testing of small study effects using Egger’s test.

Visual inspection indicated symmetry of funnel plots of the association of the risk allele with first and subsequent CHD events, confirmed by the Egger test, which provided no evidence of small-study bias (Fig. 4).

No difference in risk allele frequencies in the cohorts of individuals without prior CHD and those with established CHD was identified, arguing against presence of survival bias. Among individuals without prior CHD, the median risk allele frequencies for the 2 most widely reported SNPs at rs1333049 and rs10757278 were 0.48 and 0.49, respectively. The corresponding frequencies of these alleles in those with established CHD were almost identical at 0.47 and 0.49 (Online Table S5).

To examine index event bias, we investigated distribution of risk factors by Ch9p21 genotype. Among the 15 subsequent event studies, 6 reported covariate distributions by risk-allele genotype. Of these, only the smallest study of 496 patients reported lower prevalence of key cardiovascular risk factors in the risk allele groups (27). No difference in risk factor frequency by Ch9p21 genotype was observed in the remaining 5 larger studies reporting these data (Online Table S6). Taken together, these findings argue against small-study, survival, or index event bias accounting for the differential effect of Ch9p21 on first and subsequent CHD events.

## Discussion

In a systematic review and meta-analysis of more than 190,000 individuals, we found evidence of a differential association of genetic variants at the Ch9p21 locus with risk of first CHD events among individuals without prior CHD and subsequent events among patients with established CHD. The null effect estimate for subsequent events did not appear to be due to lack of power, nor was there evidence that the differential association arose from bias.

Genetic variants at the Ch9p21 locus have been consistently replicated in case-control studies for association with *prevalent* CHD (37). This is the first study to thoroughly investigate the association of Ch9p21 in prospective studies with *incident* events and contrast the differential effect according to whether the event was first or subsequent. Early prospective studies in individuals without prior CHD demonstrated a 15% to 35% increase in risk, per risk allele, of incident CHD over follow-up periods of up to 10 years 6, 32. In contrast, in clinical cohorts of patients with established CHD, such as those undergoing coronary angiography or those recruited following an acute coronary syndrome, the association with subsequent events has been less clear 9, 10, 11, 12, 13, 35. However, the majority of such studies have been small with heterogeneous endpoints, making it difficult to draw definitive conclusions. Using the available evidence, our meta-analysis, including more than 190,000 patients with 20,000 incident events, confirms that Ch9p21 associates robustly with risk of first events in those without prior CHD but not subsequent events in those with established CHD.

In observational studies of this nature, bias can play an important role in distorting underlying associations, although assessment of such biases is rarely considered. However, in this study, we have given due consideration to the potential for such biases to enable appropriate interpretation of our findings.

First, we found no evidence of publication bias (which might have overinflated the genetic association with first CHD events) or lack of statistical power (which might have led spuriously to null genetic effect estimate in clinical cohorts), and in careful subgroup analysis, we identified very few sources of heterogeneity and potential bias (e.g., systematic differences in age, sex distribution, SNP type, and proportion of studies with adjudicated event) as an explanation of the difference in effect estimates.

Second, the observed null association of Ch9p21 with subsequent events could be explained by survival bias, a more common concern for case-control studies where ascertainment occurs after an event, such that fatal cases are excluded (38). If individuals with the risk genotype were at disproportionate risk of fatal events, this might lead to a depletion of individuals with the Ch9p21 variant who have severe disease in clinical cohorts and thus diminish the effect estimate for the association of Ch9p21 with subsequent events. If this were present, we would expect the frequency of Ch9p21 risk alleles to be lower in the established disease cohorts compared with the population cohorts. However, we found no evidence of a difference in frequency of Ch9p21 alleles between population-based and clinical cohorts. Furthermore, computational models of case-control survival bias also suggest that the effect is minimal for exposures (or variants) with small effect sizes, as is the case for Ch9p21 (38). Nonetheless, survival bias remains an important potential source of bias that may affect studies of recurrent disease events.

Third, our analysis could be hampered by index event bias. In this scenario, among individuals who experience a first CHD event, those exposed to the Ch9p21 variant may have reduced exposure to other cardiovascular risk factors (e.g., smoking or diabetes), compared with those experiencing an event in the absence of the Ch9p21 variant. This imbalance in risk factor distribution could distort true differences in the risk of subsequent CHD events between those patients who continue to remain exposed and unexposed to the effects of the Ch9p21 variant (39). However, when individuals with established CHD from studies of >500 patients were grouped by Ch9p21 genotype status, there was no evidence of a systematic difference in traditional cardiovascular risk factors by genotype group. Furthermore, the traditional means to control for confounding is to adjust for the putative confounder in a multivariate analysis model. It is thus important to note that in most studies included in our analysis, there was comprehensive adjustment for cardiovascular covariates (Fig. 1). These 2 pieces of evidence argue against index event bias driving the null effect of Ch9p21 with subsequent CHD events.

Despite these potential sources of bias, it is tempting to consider a biological explanation to account for the observed heterogeneity of effect estimates in population and clinical cohorts. Currently, this is hampered by the limited knowledge base regarding the precise molecular mechanism by which variants at the Ch9p21 locus confer risk of CHD, especially because variants in this region are distant from the nearest protein coding gene (40). Early studies demonstrated the association of Ch9p21 with a broad mixture of both stable and unstable CHD phenotypes. It was thus uncertain whether Ch9p21 affected the upstream phenotype of atheroma development or its downstream consequence of plaque rupture and infarction, which is important because these may represent 2 distinct biological processes, with potentially separate and/or overlapping causal factors (41). Emerging biological and clinical data suggest that Ch9p21 may promote the development of atherosclerosis. For example, experimental data suggest that Ch9p21 promotes expression of nearby cyclin-dependent kinase genes, through novel regulatory mechanisms, which in turn stimulates vascular smooth muscle cell proliferation and senescence, a key feature of atherosclerosis 42, 43, 44, 45. In support of this hypothesis, we and others have shown that Ch9p21 variants are associated with: 1) angiographic CAD burden 46, 47, 48, 49; 2) subclinical atherosclerosis (50); 3) coronary calcification (51); 4) carotid atherosclerosis (6); and 5) peripheral arterial disease 6, 52. Furthermore, a recent large-scale analysis found a lack of association between Ch9p21 and MI in patients with underlying CAD in case-control datasets (41), a result that was further corroborated by meta-analysis (48). Ch9p21 may, therefore, play a more important role in gradual atherosclerosis development rather than acute plaque rupture. This could in part explain why an association was identified between the risk allele of Ch9p21 and subsequent revascularization as opposed to subsequent MI, that is, elective revascularization procedures are typically conducted in the setting of a stable atherosclerotic plaque in which there is >70% stenosis evident on angiography (reflecting build-up of atheroma), whereas MI is often heralded by plaque rupture and thrombus formation, often in the setting of nonobstructive atheroma (53).

Alternative explanations are that pharmacological therapy and/or coronary interventions following diagnosis of CHD attenuates the genetic risk associated with Ch9p21. Given that existing data suggests that Ch9p21 promotes atheroma development, it is plausible that statin therapy, which retards and potentially reverses atherosclerosis in high doses, may diminish any ongoing impact of Ch9p21 54, 55. This would be even more apparent if Ch9p21 carriers (compared with noncarriers) tended to receive greater statin doses given their higher degree of atheroma burden.

Our study has important implications for future research in this field and for clinical translation. First, our observations indicate that future genetic association studies should be more circumspect about assuming that genetic variants have similar effects for both first and subsequent CHD events. Second, important mechanistic differences may account for the differential genetic effect, and further research into this area is needed to enhance understanding of the underlying reasons. Third, these findings argue for a consortium of studies set in individuals with established CHD to better understand the genomic susceptibility to subsequent CHD events and also to perform detailed analysis, including assessment for selection biases, which is not possible with literature-based meta-analyses. Finally, these results are of importance for risk prediction using genotype data: despite studies failing to show incremental value for Ch9p21 in risk prediction models for identifying risk of *first* events (56), direct-to-consumer and physician-ordered testing remains available; our findings highlight potential pitfalls of using metrics outside of the populations from which they were derived.

### Study strengths and limitations

The primary strength of our study is the large sample size, bringing together published and unpublished data on association of Ch9p21 and incident risk. We were not able to incorporate data from 4 cohorts that reported recessive genetic models 25, 29, 31, although 3 of these did not show evidence of an association between Ch9p21 and subsequent CHD events, in support of our findings. Despite this, with more than 5-fold more individuals in the analysis of Ch9p21 with first CHD events, it is possible that our analysis of the relationship between Ch9p21 with subsequent events was underpowered. Against this argument is our power calculation, which estimated >99% power to detect an effect estimate of similar magnitude to that of Ch9p21 for first CHD events and >90% power to detect an effect estimate of one-half of the magnitude. It remains possible that Ch9p21 does associate with subsequent CHD events but with a smaller magnitude of effect, and that we were underpowered to detect it. Additionally, studies of cohorts with established CHD reported a broader array of endpoints that might dilute a true association with a particular subset of endpoints. This could be addressed in part by conducting an appropriately powered meta-analysis of studies with a range of endpoints and access to participant level rather than only summary data. A complementary approach would be to estimate associations between first and subsequent CHD events in a single large-scale cohort such as the U.K. Biobank (57), in which genetic, covariate, and outcome data are available on a mix of participants, some healthy and some with prevalent CHD collected to a common protocol.

## Conclusions

We have demonstrated through systematic review and meta-analysis that, although Ch9p21 associates strongly with risk of first events in those without prior CHD, it does not associate with risk of subsequent events in those with established CHD.
