# Supplementary material for: Genetic Variants at Chromosome 9p21 and Risk of First Versus Subsequent Coronary Heart Disease Events: A Systematic Review and Meta-Analysis
Source: J Am Coll Cardiol. 2014 Jun 3;63(21):2234–45. doi: 10.1016/j.jacc.2014.01.065 (PMC4035794; doi:10.1016/j.jacc.2014.01.065)
Supplement: Online Data [file mmc1.doc]

# Supplementary online material

# Tables

## Table S1. Genomic characteristics of the included studies

| **Source (First author, cohort name, reference)** | **SNP used to genotype Ch9p21** | **Genotype platform (Chip, PCR)** | **Call rate, %** | **Hardy Weinberg Equilibrium**  **P-value** |
| --- | --- | --- | --- | --- |
| **Studies reporting first coronary events in individuals without prior CHD** | | | | |
| Vaarhorst, CAREMA[^1^](#_ENREF_1) | rs10757278 | Chip (Sequenom mass array) | 98.4 | 0.61 |
| Ye, Bruneck[^2^](#_ENREF_2) | rs1333049 | PCR (Taqman) | NR | 0.41 |
| Dutta, EPES[^3^](#_ENREF_3) | rs1333049 | PCR (Taqman) | NR | >0.05 |
| Lluis-Ganella, REGICOR [^4^](#_ENREF_4) | rs1333049 | Chip (CardioChip ) | NR | 0.2 |
| Talmud, Northwick Park [^5^](#_ENREF_5) | rs10757274 | PCR (Taqman) | NR | NR |
| Lluis-Ganella, Framingham [^4^](#_ENREF_4) | rs1333049 | Chip (Affymetrix 500k) | NR | 1 |
| Franceschini, CHS [^6^](#_ENREF_6) | rs10757278 | PCR (Taqman) | NR | NR |
| Wahlstrand, NORDIL [^7^](#_ENREF_7) | rs10757278 | Chip (Sequenom mass array) | 98.7 | >0.1 |
| Dehgan, Rotterdam [^8^](#_ENREF_8) | rs10757278 | PCR (Taqman) | NR | NR |
| Franceschini, ARIC [^6^](#_ENREF_6) | rs10757278 | PCR (Taqman) | NR | NR |
| Mcpherson, Copenhagen City Heart Study [^9^](#_ENREF_9) | rs10757274 | PCR (Taqman) | NR | 0.56 |
| Franceschini, Women’s Health Initiative [^6^](#_ENREF_6) | rs10757278 | Chip (Illumina golden gate) | >95 | NR |
| Paynter, Women's Health Study[^10^](#_ENREF_10) | rs10757274 | PCR (Taqman) | NR | NR |
| Tikkanen, FINRISK/Health2000 [^11^](#_ENREF_11) | rs4977574 | Chip (Sequenom mass array) | 98 | NR |
| Gransbo, MALMO DCS [^12^](#_ENREF_12) | rs4977574 | PCR (Taqman) | NR | 0.48 |
| Karvanen, MORGAM [^13^](#_ENREF_13) | rs1333049 | Chip (Sequenom mass array) | NR | NR |
| **Studies reporting subsequent coronary events in individuals with established CHD** | | | | |
| Ellis, PMI [^14^](#_ENREF_14) | rs1333049 | PCR (Taqman) | NR | 0.27 |
| Ardissino, IGSEMI [^15^](#_ENREF_15) | rs1333040 | Chip (Sequenom mass array) | 99.8 | >0.05 |
| Virani, TexGEn [^16^](#_ENREF_16) | rs1333049 | PCR (Taqman) | >99 | >0.05 |
| Wauters, GRACE [^17^](#_ENREF_17) | rs4977574 | Chip (Sequenom mass array) | 99.6 | >0.05 |
| Dutta, EPES[^3^](#_ENREF_3) | rs10757278 | PCR (Taqman) | NR | >0.05 |
| Pereira, MASS II [^3^](#_ENREF_3) | rs10757278 | PCR | 95 | 0.64 |
| Andreassi, GENECOR [^18^](#_ENREF_18) | rs1333049 | PCR (LC green light scanner) | NR | NR |
| Gong, INFORM [^19^](#_ENREF_19) | rs10757278 | PCR (Taqman) | NR | 0.66 |
| Ellis, CDCS [^14^](#_ENREF_14) | rs1333049 | PCR (Taqman) | NR | 0.02 |
| Virani, TexGen [^16^](#_ENREF_16) | rs1333049 | PCR (Taqman) | >99 | >0.05 |
| Hoppmann, German Stent Study [^20^](#_ENREF_20) | rs1333049 | PCR (Taqman) | NR | NR |
| Gong, INVEST-GENES [^19^](#_ENREF_19) | rs10757278 | Chip (Illumina 50K) | >95 | 0.79 |
| Patel, Emory, unpublished | rs10757278 | PCR (SNPstream) | NR | 0.5 |
| Patel, Cleveland Clinic [^21^](#_ENREF_21) | rs10757278 | Chip (Affymetrix 6.0) | NR | 1 |
| Horne, Intermountain 1A [^22^](#_ENREF_22) | rs2383206 | PCR (Taqman) | NR | NR |
| Horne, Intermountain 1B [^22^](#_ENREF_22) | rs2383206 | PCR (Taqman) | NR | NR |
| Asselbergs, SMART, unpublished | rs1333049 | PCR (Taqman) | 97.3 | 0.79 |

**Abbreviations**. CHD: Coronary Heart Disease; NR: not reported; PCR: polymerase chain reaction.

## Table S2. Documentation of risk allele used for the analysis of the association of 9p21 SNPs with coronary events

| **SNP rs#** | **Risk allele** | **Studies (first author)** |
| --- | --- | --- |
| rs10757278 | G | Franceschini, Wahlstrand, Patel, Patel, Gong, Pereira, Vaarhorst |
| rs1333049 | C | Ye, Karvanen, Ganella, Dutta, Virani, Hoppman, Anreassi |
| rs4977574 | G | Tikanen, Gransbo, Paynter, Wauters, Asselbergs |
| rs10757274 | G | Talmud, McPherson |
| rs133040 | T | Ardissino |
| rs2383206 | G | Horne |

## Table S3. Heterogeneity in the association of Ch9p21 (per risk allele) with subsequent coronary events before and after adjusting for covariates

| **Adjustment** | ***I*^2^, %** | **Heterogeneity**  **Category** § |
| --- | --- | --- |
| Unadjusted | 64 | Moderate |
| SNP rs# | 33 | Low |
| Mean age | 53 | Moderate |
| Genotype platform | 48 | Low |
| SNP rs#, mean age, genotype platform | 41 | Low |

**Footnote**: § heterogeneity category derived from Higgins et al [^23^](#_ENREF_23)

## Table S4. Study contributions to individual and composite outcomes

| **Source (First author, cohort name, reference)** | **All-cause mortality** | **MI, death, unstable angina, revascularization, hospitalization, peripheral arterial disease** | **MI, all-cause mortality, unstable angina, revascularization, hospitalization** | **Fatal/nonfatal MI** | **Revascularization** | **Coronary Events Composite** |
| --- | --- | --- | --- | --- | --- | --- |
| **Studies set in individuals without prior CHD** | | | | | | |
| Franceschini, ARIC [^6^](#_ENREF_6) |  |  | • | • |  | • |
| Ye, Bruneck[^2^](#_ENREF_2) |  | • | • |  |  | • |
| Vaarhorst, CAREMA[^1^](#_ENREF_1) |  |  | • |  |  | • |
| Mcpherson, Copenhagen City Heart Study [^9^](#_ENREF_9) |  | • |  |  |  | • |
| Franceschini, CHS [^6^](#_ENREF_6) |  | • | • | • |  | • |
| Dutta, EPES[^3^](#_ENREF_3) | • |  |  | • |  | • |
| Tikkanen, FINRISK/Health2000 [^11^](#_ENREF_11) |  |  | • |  |  | • |
| Lluis-Ganella, Framingham [^4^](#_ENREF_4) |  |  | • |  |  | • |
| Gransbo, MALMO DCS [^12^](#_ENREF_12) |  |  | • |  |  | • |
| Karvanen, MORGAM [^13^](#_ENREF_13) | • |  | • |  |  | • |
| Wahlstrand, NORDIL [^7^](#_ENREF_7) |  |  | • |  |  | • |
| Talmud, Northwick Park [^5^](#_ENREF_5) |  |  | • |  |  | • |
| Lluis-Ganella, REGICOR [^4^](#_ENREF_4) |  |  | • |  |  | • |
| Dehgan, Rotterdam [^8^](#_ENREF_8) |  |  | • | • |  | • |
| Franceschini, Women’s Health Initiative [^6^](#_ENREF_6) |  |  | • | • |  | • |
| Paynter, Women's Health Study[^10^](#_ENREF_10) |  |  | • | • |  | • |
| **Studies set in individuals with established CHD** | | | | | | |
| Patel, Cleveland Clinic [^21^](#_ENREF_21) |  |  |  | • |  | • |
| Dutta, EPES [**^3^**](#_ENREF_3) | • |  |  | • |  | • |
| Patel, Emory, unpublished | • |  | • | • | • | • |
| Andreassi, GENECOR [^18^](#_ENREF_18) |  |  | • |  |  | • |
| Wauters, GRACE [^17^](#_ENREF_17) |  |  |  | • |  | • |
| Hoppmann, German Stent Study [^20^](#_ENREF_20) |  |  | • |  |  | • |
| Ardissino, IGSEMI [^15^](#_ENREF_15) |  |  | • | • | • | • |
| Gong, INFORM [^19^](#_ENREF_19) |  |  | • |  |  | • |
| Gong, INVEST-GENES [^19^](#_ENREF_19) |  |  |  |  |  | • |
| Horne, Intermountain 1A [^22^](#_ENREF_22) |  |  |  |  |  | • |
| Horne, Intermountain 1B [^22^](#_ENREF_22) |  |  |  |  |  | • |
| Pereira, MASS II [^3^](#_ENREF_3) | • |  |  |  |  | • |
| Asselbergs, SMART, unpublished |  |  | • | • |  | • |
| Virani, TexGEn [^16^](#_ENREF_16) **(**ACS**)** |  |  |  | • |  | • |
| Virani, TexGEn [^16^](#_ENREF_16)**(**CABG**)** |  |  |  | • |  | • |

**Abbreviations.** ACS: acute coronary syndrome; CAD: coronary artery disease; MI: myocardial infarction.

## Table S5. Median 9p21 risk allele frequencies in first event versus subsequent event studies

| **SNP** | **Number of studies, allele frequency** | |
| --- | --- | --- |
| Rs number | First events | Subsequent events |
| rs1333049 | 5, 0.48 | 4, 0.47 |
| rs10757278 | 6, 0.49 | 5, 0.49 |

## Table S6. Risk factor distribution by Ch9p21 genotype in studies of patients with established CHD

| **Source (First author, cohort name, reference)** | **CVD Risk Factor prevalence reported by Ch9p21 genotype** | **Reported differences** |
| --- | --- | --- |
| Dutta, EPES[^3^](#_ENREF_3) | Not reported | N/A |
| Pereira, MASS II [^3^](#_ENREF_3) | age, gender, BMI, HL, smoking | HT and DM less frequent in risk genotypes |
| Virani, TexGen [^16^](#_ENREF_16) (CABG) | Not reported | N/A |
| Andreassi, GENECOR [^18^](#_ENREF_18) | Not reported | N/A |
| Patel, Cleveland Clinic [^21^](#_ENREF_21) | Not reported | N/A |
| Gong, INFORM [^19^](#_ENREF_19) | Not reported | N/A |
| Gong, INVEST-GENES [^19^](#_ENREF_19) | Not reported | N/A |
| Virani, TexGen [^16^](#_ENREF_16) (ACS) | Not reported | N/A |
| Wauters, GRACE [^17^](#_ENREF_17) | age, gender, BMI, FH, T2D, smoking HT, HL | No differences across genotypes |
| Asselbergs, SMART, unpublished | Not reported | N/A |
| Hoppmann, German Stent Study [^20^](#_ENREF_20) | Not reported | N/A |
| Ardissino, IGSEMI [^15^](#_ENREF_15) | age, gender, FH, T2D, smoking, HT, BMI, HL | No differences across genotypes |
| Patel, Emory, unpublished | age, gender, FH, T2D, smoking, HT, BMI, HL (unpublished) | No differences across genotypes |
| Horne, Intermountain 1A [^22^](#_ENREF_22) | age, gender, FH, T2D, smoking, HT, HL, BMI | No differences across genotypes |
| Horne, Intermountain 1B [^22^](#_ENREF_22) | age, gender, FH, T2D, smoking, HT, HL, BMI | No differences across genotypes |

**Abbreviations**: BMI: body mass index; FH: family history of CHD; HT: hypertension; HL: hyperlipidemia; N/A: not available; T2D: type 2 diabetes.

Figures

## Figure S1. Identification of articles for the systematic review and meta-analysis


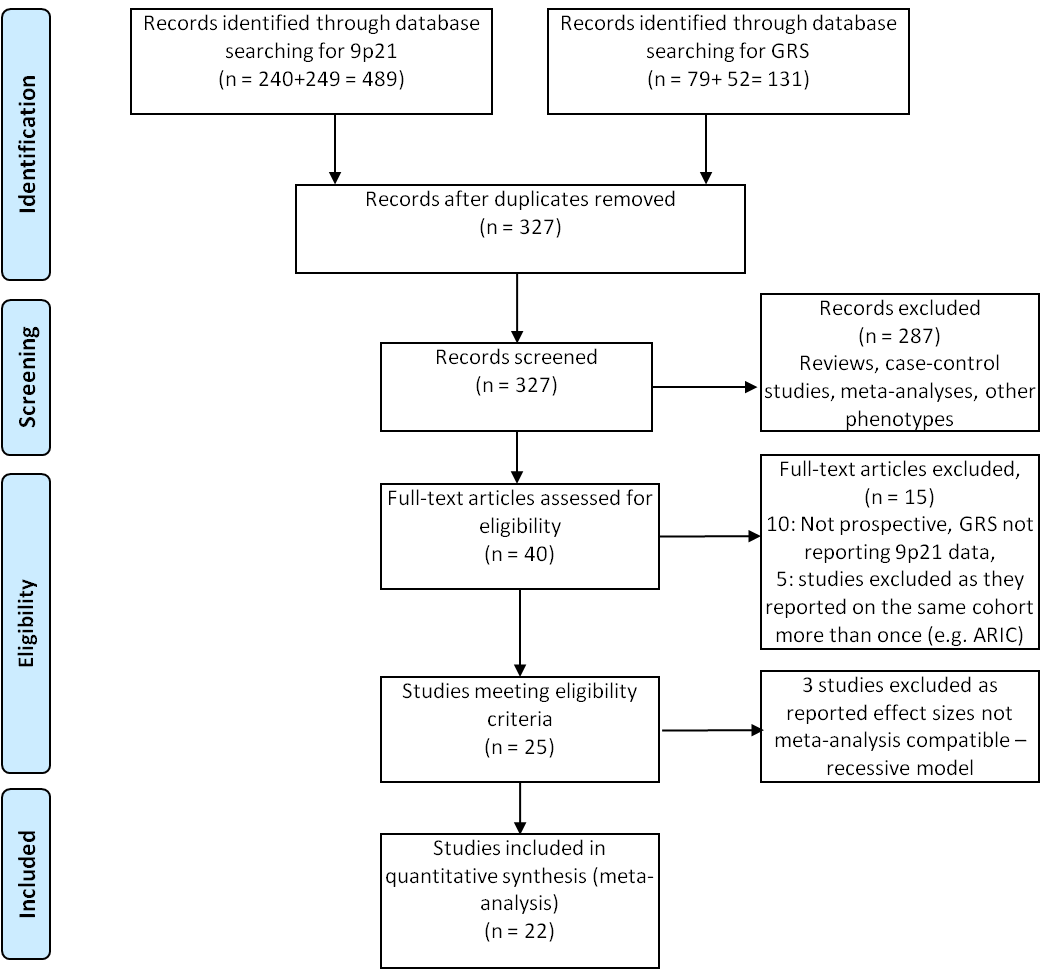


## Figure S2. Linkage disequilibrium plot of the SNPs used to genotype Ch9p21


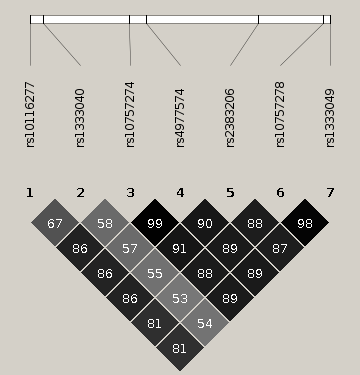


**Footnote**: values in boxes represent linkage disequilibrium between SNPs as quantified using R^2^.

## Figure S3. Influence of removing each study on the summary estimate of the association of Ch9p21 risk allele with first and subsequent coronary events

**First Coronary Events**

**
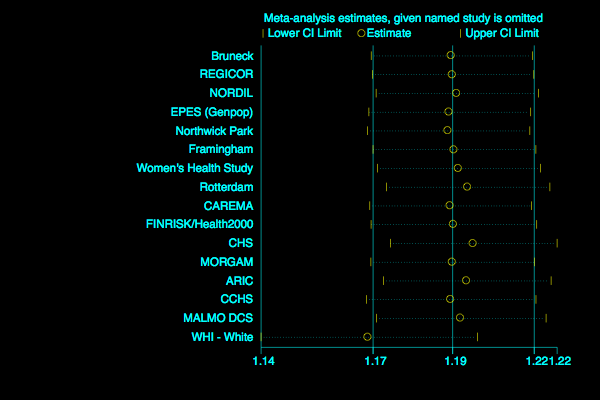
**

**Subsequent Coronary Events**

**
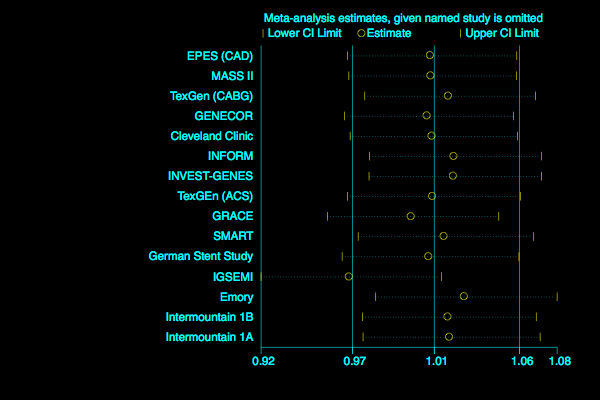
**

**Footnote**: Circles are point estimates and vertical lines 95%CI for the summary hazards ratio when each study is omitted from the meta-analysis.

## Figure S4. Association of Ch9p21 (per risk allele) with first and subsequent coronary event in different ethnic groups


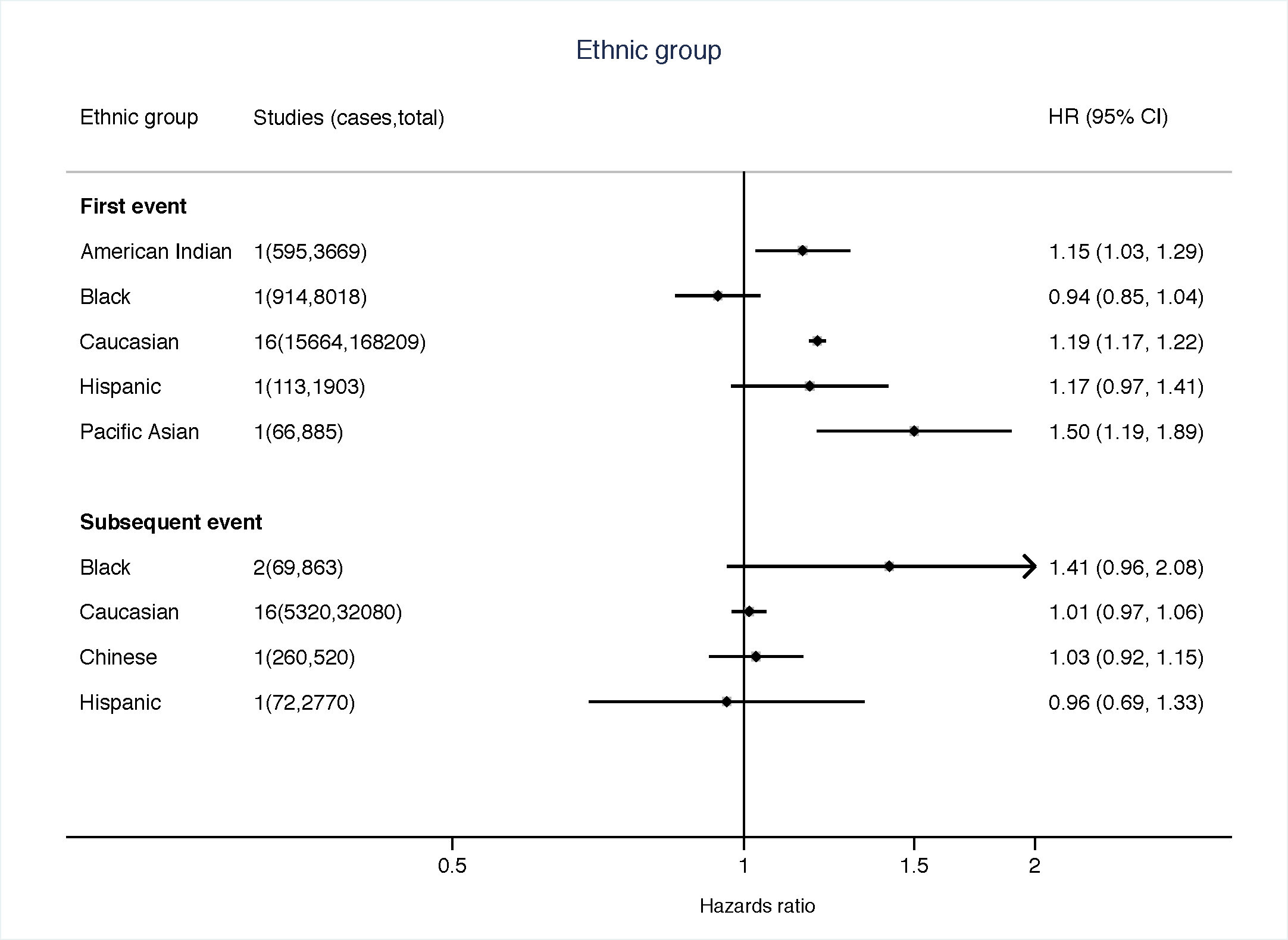


# eMethods

## Search Strategy

### PubMed

The search terms used in PubMed were:

**(1) 9p21 and CHD**

(9p21[All Fields] OR 9p21.3[All Fields]) AND (("heart"[MeSH Terms] OR "heart"[All Fields] OR "coronary"[All Fields]) OR ("cardiovascular system"[MeSH Terms] OR ("cardiovascular"[All Fields] AND "system"[All Fields]) OR "cardiovascular system"[All Fields] OR "cardiovascular"[All Fields]) OR ("myocardium"[MeSH Terms] OR "myocardium"[All Fields] OR "myocardial"[All Fields]))

**(2) Genetic risk score and CHD**

("genetic risk score"[All Fields]) AND (("heart"[MeSH Terms] OR "heart"[All Fields] OR "coronary"[All Fields]) OR ("cardiovascular system"[MeSH Terms] OR ("cardiovascular"[All Fields] AND "system"[All Fields]) OR "cardiovascular system"[All Fields] OR "cardiovascular"[All Fields]) OR ("myocardium"[MeSH Terms] OR "myocardium"[All Fields] OR "myocardial"[All Fields]))

### EMBASE

The search terms used in EMBASE were:

**(1) 9p21 and CHD**

(9p21 and (cardiovascular or coronary or myocardial)).mp. [mp=title, abstract, subject headings, heading word, drug trade name, original title, device manufacturer, drug manufacturer, device trade name, keyword]

***-****Search terms used:*

- 9p21
- cardiovascular
- coronary
- myocardial

**(2) Genetic risk score and CHD**

(Genetic Risk Score and (Coronary or Myocardial or cardiovascular)).mp. [mp=title, abstract, subject headings, heading word, drug trade name, original title, device manufacturer, drug manufacturer, device trade name, keyword]

***-****Search terms used:*

- cardiovascular
- coronary
- genetic
- genetic risk score
- myocardial
- risk
- score

# References

1. Vaarhorst AA, Lu Y, Heijmans BT, et al. Literature-based genetic risk scores for coronary heart disease: the Cardiovascular Registry Maastricht (CAREMA) prospective cohort study. *Circulation. Cardiovascular genetics.* Apr 1 2012;5(2):202-209.

2. Ye S, Willeit J, Kronenberg F, Xu Q, Kiechl S. Association of genetic variation on chromosome 9p21 with susceptibility and progression of atherosclerosis: a population-based, prospective study. *Journal of the American College of Cardiology.* Jul 29 2008;52(5):378-384.

3. Dutta A, Henley W, Lang IA, et al. The coronary artery disease-associated 9p21 variant and later life 20-year survival to cohort extinction. *Circulation. Cardiovascular genetics.* Oct 2011;4(5):542-548.

4. Lluis-Ganella C, Subirana I, Lucas G, et al. Assessment of the value of a genetic risk score in improving the estimation of coronary risk. *Atherosclerosis.* Jun 2012;222(2):456-463.

5. Talmud PJ, Cooper JA, Palmen J, et al. Chromosome 9p21.3 coronary heart disease locus genotype and prospective risk of CHD in healthy middle-aged men. *Clinical chemistry.* Mar 2008;54(3):467-474.

6. Franceschini N, Carty C, Buzkova P, et al. Association of genetic variants and incident coronary heart disease in multiethnic cohorts: the PAGE study. *Circulation. Cardiovascular genetics.* Dec 2011;4(6):661-672.

7. Wahlstrand B, Orho-Melander M, Delling L, et al. The myocardial infarction associated CDKN2A/CDKN2B locus on chromosome 9p21 is associated with stroke independently of coronary events in patients with hypertension. *Journal of hypertension.* Apr 2009;27(4):769-773.

8. Dehghan A, van Hoek M, Sijbrands EJ, et al. Lack of association of two common polymorphisms on 9p21 with risk of coronary heart disease and myocardial infarction; results from a prospective cohort study. *BMC medicine.* 2008;6:30.

9. McPherson R, Pertsemlidis A, Kavaslar N, et al. A common allele on chromosome 9 associated with coronary heart disease. *Science.* Jun 8 2007;316(5830):1488-1491.

10. Paynter NP, Chasman DI, Buring JE, Shiffman D, Cook NR, Ridker PM. Cardiovascular disease risk prediction with and without knowledge of genetic variation at chromosome 9p21.3. *Annals of internal medicine.* Jan 20 2009;150(2):65-72.

11. Tikkanen E, Havulinna AS, Palotie A, Salomaa V, Ripatti S. Genetic Risk Prediction and a 2-Stage Risk Screening Strategy for Coronary Heart Disease. *Arteriosclerosis, thrombosis, and vascular biology.* Apr 18 2013.

12. Gransbo K, Almgren P, Sjogren M, et al. Chromosome 9p21 genetic variation explains 13% of cardiovascular disease incidence but does not improve risk prediction. *Journal of internal medicine.* Mar 8 2013.

13. Karvanen J, Silander K, Kee F, et al. The impact of newly identified loci on coronary heart disease, stroke and total mortality in the MORGAM prospective cohorts. *Genetic epidemiology.* Apr 2009;33(3):237-246.

14. Ellis KL, Pilbrow AP, Frampton CM, et al. A common variant at chromosome 9P21.3 is associated with age of onset of coronary disease but not subsequent mortality. *Circulation. Cardiovascular genetics.* Jun 2010;3(3):286-293.

15. Ardissino D, Berzuini C, Merlini PA, et al. Influence of 9p21.3 genetic variants on clinical and angiographic outcomes in early-onset myocardial infarction. *Journal of the American College of Cardiology.* Jul 19 2011;58(4):426-434.

16. Virani SS, Brautbar A, Lee VV, et al. Chromosome 9p21 single nucleotide polymorphisms are not associated with recurrent myocardial infarction in patients with established coronary artery disease. *Circulation journal : official journal of the Japanese Circulation Society.* 2012;76(4):950-956.

17. Wauters E, Carruthers KF, Buysschaert I, et al. Influence of 23 coronary artery disease variants on recurrent myocardial infarction or cardiac death: the GRACE Genetics Study. *European heart journal.* Apr 2013;34(13):993-1001.

18. Andreassi MG, Adlerstein D, Carpeggiani C, et al. Individual and summed effects of high-risk genetic polymorphisms on recurrent cardiovascular events following ischemic heart disease. *Atherosclerosis.* Aug 2012;223(2):409-415.

19. Gong Y, Beitelshees AL, Cooper-DeHoff RM, et al. Chromosome 9p21 haplotypes and prognosis in white and black patients with coronary artery disease. *Circulation. Cardiovascular genetics.* Apr 2011;4(2):169-178.

20. Hoppmann P, Erl A, Turk S, et al. No association of chromosome 9p21.3 variation with clinical and angiographic outcomes after placement of drug-eluting stents. *JACC. Cardiovascular interventions.* Nov 2009;2(11):1149-1155.

21. Patel RS, Sun YV, Hartiala J, et al. Association of a genetic risk score with prevalent and incident myocardial infarction in subjects undergoing coronary angiography. *Circulation. Cardiovascular genetics.* Aug 1 2012;5(4):441-449.

22. Horne BD, Carlquist JF, Muhlestein JB, Bair TL, Anderson JL. Association of variation in the chromosome 9p21 locus with myocardial infarction versus chronic coronary artery disease. *Circulation. Cardiovascular genetics.* Dec 2008;1(2):85-92.

23. Higgins JP, Thompson SG, Deeks JJ, Altman DG. Measuring inconsistency in meta-analyses. *Bmj.* Sep 6 2003;327(7414):557-560.
